# An Experimental Realization of a Chaos-Based Secure Communication Using Arduino Microcontrollers

**DOI:** 10.1155/2015/123080

**Published:** 2015-08-27

**Authors:** Mauricio Zapateiro De la Hoz, Leonardo Acho, Yolanda Vidal

**Affiliations:** ^1^Universidade Tecnológica Federal do Paraná, Avenida Alberto Carazzai 1640, 86300-000 Cornélio Procópio, PR, Brazil; ^2^Control, Dynamics and Applications Group (CoDAlab), Departament de Matemàtica Aplicada III, Universitat Politécnica de Catalunya, d'Urgell 187, E08036 Barcelona, Spain

## Abstract

Security and secrecy are some of the important concerns in the communications world. In the last years, several encryption techniques have been proposed in order to improve the secrecy of the information transmitted. Chaos-based encryption techniques are being widely studied as part of the problem because of the highly unpredictable and random-look nature of the chaotic signals. In this paper we propose a digital-based communication system that uses the logistic map which is a mathematically simple model that is chaotic under certain conditions. The input message signal is modulated using a simple Delta modulator and encrypted using a logistic map. The key signal is also encrypted using the same logistic map with different initial conditions. In the receiver side, the binary-coded message is decrypted using the encrypted key signal that is sent through one of the communication channels. The proposed scheme is experimentally tested using Arduino shields which are simple yet powerful development kits that allows for the implementation of the communication system for testing purposes.

## 1. Introduction

Security and secrecy in communications are some of the most important concerns in societies nowadays. With the advent of worldwide networks and digital communication techniques, the cryptographic techniques that once were restricted to military and state affairs are now covering several domains such as banks, private companies, medical organizations, and so forth. This has led to a very active research field oriented to finding optimal solutions to the problem of communications security [1–3]. As a result, numerous cryptographic techniques that seek to preserve the privacy of the information transmitted have been designed. Chaos is the base of many encryption and decryption techniques because chaotic signals have a highly unpredictable and random-look nature [4].

There are basically two main approaches to designing secure communication systems based on chaotic dynamics: analog and digital. Analog communication systems based on chaos are possible because of the possibility of synchronization [5]. Synchronization occurs when the output of the driving system (master) controls the response system (slave) in such a way that they both oscillate in a synchronized manner. On the other hand, digital chaos communication systems do not depend on chaos synchronization at all. Instead, they usually use one or more chaotic maps in which the initial conditions and the control parameters play the role of the secret key [6].

Several examples of chaos-based communication systems can be found in the literature. For instance, Zapateiro et al. [7] designed a chaotic communication system in which a binary signal is encrypted in the frequency of the sinusoidal term of a chaotic Duffing oscillator. Two chaotic signals of the oscillator are further encrypted with a Delta modulator before they are sent through the channel. In the receiver, a Lyapunov-based observer uses the chaotic signals for retrieving the sinusoidal term that contains the message. A novel frequency estimator is then used to obtain the binary signal. Furthermore, in a new proposal, Zapateiro De la Hoz et al. [8] investigated a modified Chua chaotic oscillator in which the nonlinear term of the original oscillator was changed for a smooth and bounded function that allows for easier analysis and synchronization with other oscillators. An application to secure communications using the modified oscillator was developed and its performance evaluated by numerical simulations. Hammami [9] proposed an image cryptosystem that makes use of hyperchaotic systems. Synchronization was achieved by assuming some structural assumptions of the master system and using some aggregation techniques associated with the arrow form matrix. Fallahi and Leung [10] developed a chaotic communication system based on a chaos multiplication modulator that encrypts the signal. The chaotic signal is generated by using the Genesio-Tesi chaotic system. The authors also prove that the system security could not be broken with the existing methods at that time. Liu and Sun [11] propose a new design of chaotic cryptosystems in which they use high dimensional chaotic maps along with some cryptography techniques to achieve a high security level. The high dimensionality of the map leads to a high complexity and effective byte confusion and diffusion of the output ciphertext at the time that the small key space problem is overcome. Pareek et al. [12] designed an image encryption scheme in which two logistic maps are used along with an 80-bit key to encrypt/decrypt the images. Eight different types of operation are used to encrypt the pixels of an image; the type of operation is chosen according to the outcome of the logistic maps. This secure communication scheme was criptanalyzed in detail in Li et al. [13]. Lee et al. [14] proposed a chaotic cipher stream, a new scheme for generating pseudorandom numbers based on the composition of chaotic maps. The method consists of using one chaotic map to generate a sequence of pseudorandom bytes and then apply some permutation on them using another chaotic map. Shyamsunder and Kaliyaperumal [15] incorporate the concept of modular arithmetic and chaotic maps for image encryption and decryption. Zhang et al. [16] propose a simple but secure chaotic cipher by improving the familiar permutation-diffusion structure.

Numerous works can be found in the literature that use the logistic map for improving security in communications. The logistic map is a nonlinear discrete map originally used for modeling population growth of different species as well as economic and political phenomena [17–19]. However, under certain conditions it exhibits a chaotic behavior [20]. This characteristic has been exploited in cryptography ever since. For example, Murillo-Escobar et al. [21] presented a symmetric text cipher in which they used a 128-bit secret key, two logistic maps with optimized pseudorandom sequences, plain text characteristics, and one permutation diffusions round. Volos et al. [22] presented a chaotic random bit generator and implemented it in an Arduino board. The microcontroller runs side by side two logistic maps working in different chaotic regimes due to the different initial conditions and system parameters. Statistical tests were carried out to prove security against intruders. Pande and Zambreno [23] presented another experimental realization of a chaotic encryption scheme, this time using a Xilin Virtex 6 FPGA. They implemented a modified logistic map that improves the performance of the logistic map in terms of Lyapunov exponent and uniformity of the bifurcation diagram. Other proposals can be found in Lawrance and Wolff [24]; Chang [25]; and Singh and Sinha [26].

In this paper we present a digital chaos communication system in which the logistic map is used to encrypt the message and key of the transmission. A simple Delta modulator is used along with one of the chaotic maps to encrypt the message. The Delta modulation technique is one of the most simple and robust methods of analog-to-digital (ADC) schemes requiring serial digital communications of analog signals [27]. In this work, the transmitter and receiver are implemented in low cost, small but powerful microcontroller boards: Arduino Uno R3 [28]. The Arduino transmitter receives a message which is analog in nature and encrypts it using a logistic map and the Delta modulator. Then the Arduino receiver decrypts the message and converts it to digital form which corresponds to the Delta-modulated signal. In order to obtain the analog version of the message signal, an analog circuitry performs the demodulation and retrieves the message.

This paper is organized as follows.  Section 2 describes the problem to be treated and a scheme of the proposed solution.  Section 3 is a brief introduction to the logistic map and its applications to secure communications.  Section 4 presents the details of the implementation of the proposed technique. Finally the conclusions are presented in Section 5.

## 2. Problem Statement

**Figure 1:**
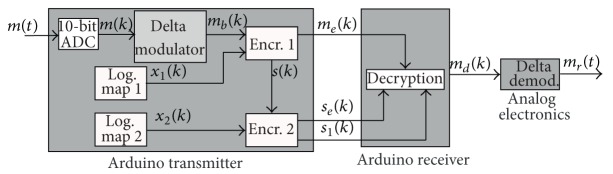
Block diagram of the communication system.

The objective of this paper is to design and implement a communication system to transmit a message *m*(*t*) between two points. The goal is to use the logistic map to encrypt the information as a security means. The proposed communication system scheme is shown in Figure 1 and it consists of the following blocks:

(i) *Arduino Transmitter.* This is the core of the transmitter. The Arduino board will take the message *m*(*t*) through one of its analog input ports. The Arduino will sample the analog input message, *m*(*t*), and convert it to the sampled signal *m*(*k*), *k* = *nT*; *T* is the sampling time, *n* = 0,1, 2,…. This signal is then encrypted by using a logistic map and a simple Delta modulator. Afterwards, a key signal, *s*(*k*), is generated in order to decrypt the message in the receiver. This key signal is further encrypted using a second logistic map. As a result, the Arduino transmitter generates three outputs: the first one is the encrypted message, *m*_*e*_(*k*), the second one is the encrypted key signal, *s*_*e*_(*k*), and the third one is an auxiliary key signal, *s*_1_(*k*), that is used for decryption purposes.
(ii) *Channels.* Three wired channels are used to send the encrypted message and key signals.
(iii) *Arduino Receiver.* This is one of the two main blocks in the receiver side. It takes the signals *m*_*e*_(*k*), *s*_*e*_(*k*), and *s*_1_(*k*) to decrypt the Delta-modulated signal before it is converted into its analog form. The output is a digital signal, *m*_*d*_(*k*), which corresponds to the signal *m*_*b*_(*k*).
(iv) *Delta Demodulator.* This is the second block in the receiver. It is a Delta demodulator consisting of an integrator, a filter, and some amplifiers to retrieve the original message. Its output is a signal *m*_*r*_(*t*) that approximates the original signal *m*(*t*).

The details of these blocks will be outlined in the following sections of this chapter.

## 3. The Logistic Map

The logistic map has its origins in the works by the Belgian mathematician Pierre-François Verhulst in the first half of the 18th century [29, 30]. Verhulst published in 1845 and 1847 two articles on how the population growth could be mathematically modeled. He called this model the logistic curve [31, 32], and it is the continuous time version of what nowadays is known as the logistic map.

**Figure 2:**
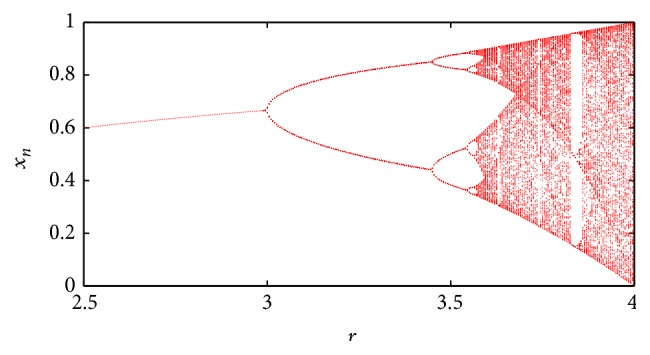
Logistic map bifurcation diagram.

The logistic map, the discrete-time version of Verhulst's logistic model, is chaotic under certain conditions. Its equation is

(1) $$\begin{array}{c} x_{i+1}=rx_{i}\left(1-x_{i}\right),\;0\leq x_{i}\leq 1, \end{array}$$

where *r* is a constant parameter.  Figure 2 is the bifurcation diagram of the logistic map created by varying the parameter *r* from 2.5 to 4.0.

**Figure 3:**
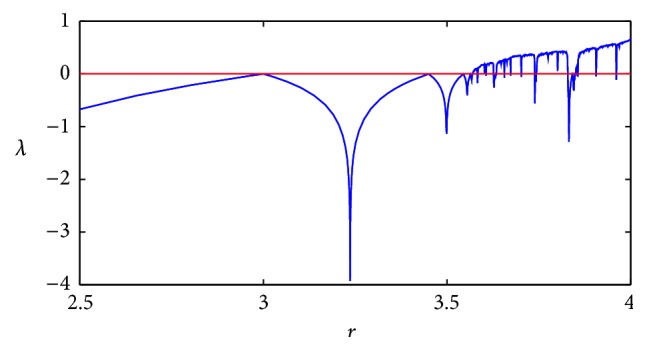
Logistic map Lyapunov exponent.

As can be seen in the bifurcation diagram, there are different regions that depend on the value of *r*. It is of particular interest when *r* = 3 because there it begins the period doubling that leads to the chaotic dynamics when *r* ≈ 3.5699… until *r* = 4.0.  Figure 3 shows the Lyapunov exponent of the logistic map as *r* is varied from 2.5 to 4.0. It can be seen that the Lyapunov exponent, *λ*, becomes positive for values of approximately greater than 3.56 which is a strong indicator of chaos [33].

In the next sections, we will use a logistic map as part of an encryption/decryption scheme for transmitting information. We will explain the details of the prototype of this communication system which is implemented on two Arduino Uno boards.

## 4. Experimental Implementation

### 4.1. Description of the Communication System

The communication system implemented in this work consists of a transmitter and a receiver whose cores are the Arduino Uno R3 microcontroller boards [28]. These are low cost, simple but powerful microcontrollers based on the ATmega328 chip. They have 14 digital input/output pins (6 of them can be used as PWM outputs), 6 analog inputs, a 16 MHz crystal oscillator, a USB connection, and a reset button. They can be programmed using a language similar to C++ called Wiring.

**Figure 4:**
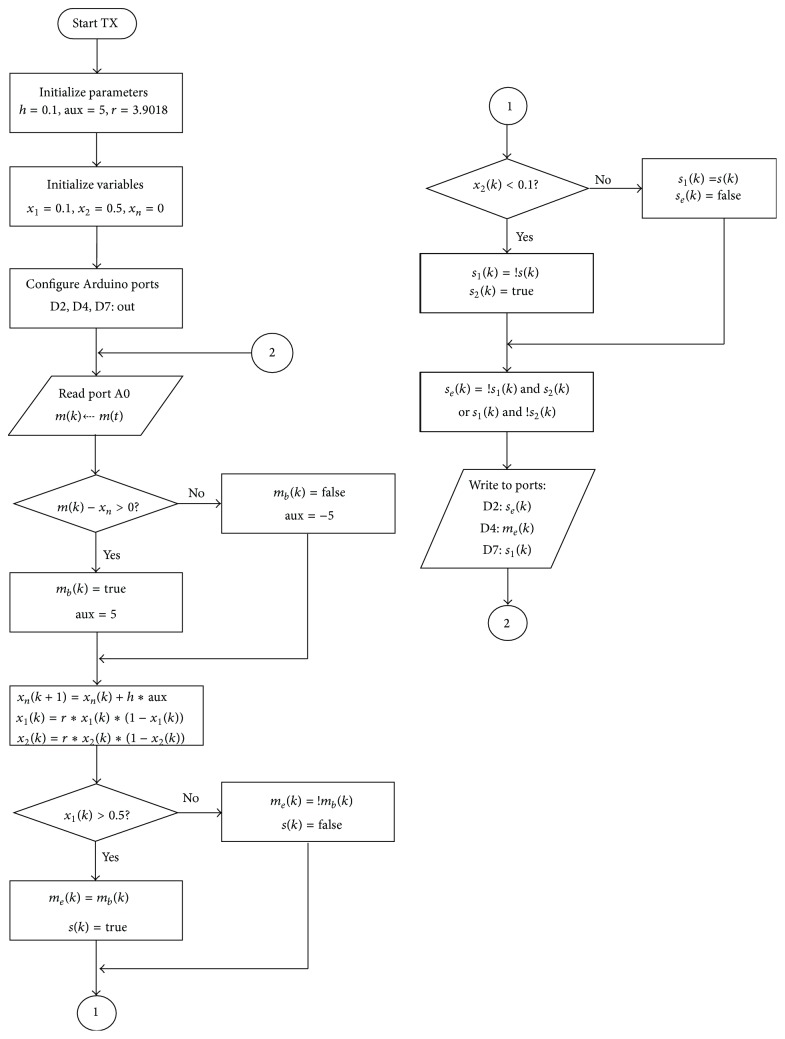
Flow diagram of the transmitter Arduino codes.

**Figure 5:**
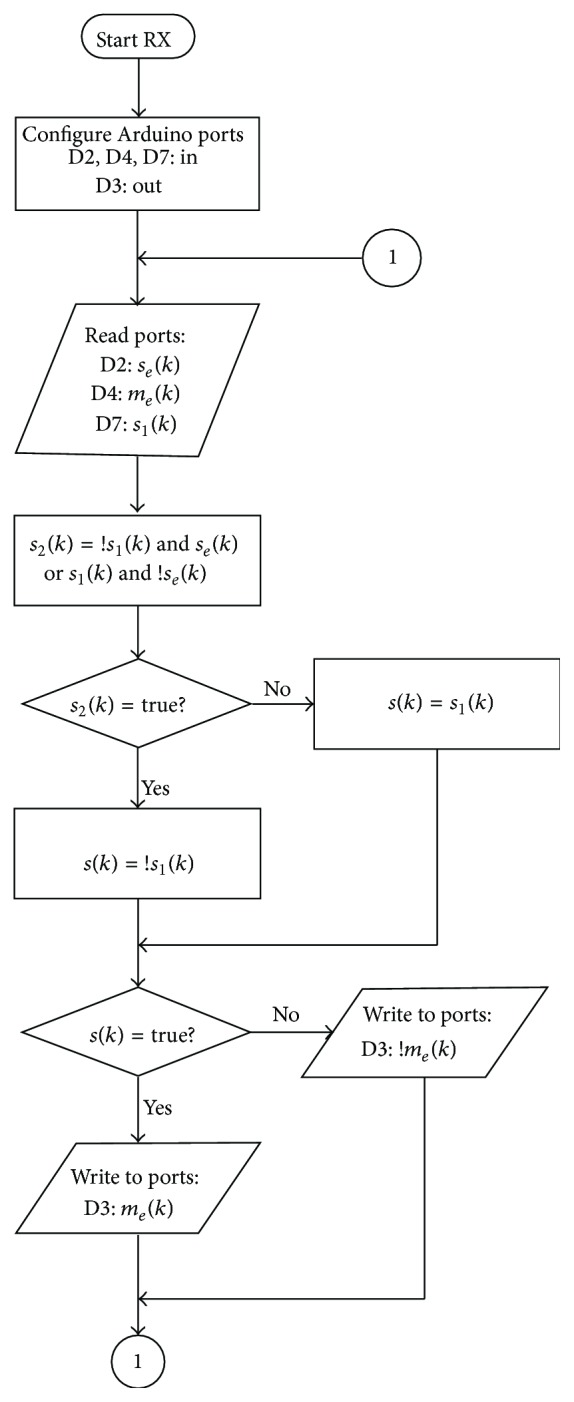
Flow diagram of the receiver Arduino codes.

The flow diagram of the programs executed by each Arduino is shown in Figures 4 and 5 in order to facilitate the description of the communication system algorithms.

The communication begins when a message *m*(*t*), generated by a function generator, and is sent to the analog input A0 of the Arduino transmitter. Arduino analog inputs only accept unipolar signals in the range from 0 V to 5 V. An embedded 10-bit ADC converts the input signal from analog to digital at a maximum rate of 10,000 samples per second. Since the output of the ADC is a value between 0 and 1023 (the ADC resolution), an internal operation to bring it back to the range from 0 V to 5 V is executed. The result is the sampled message signal *m*(*k*).

**Figure 6:**
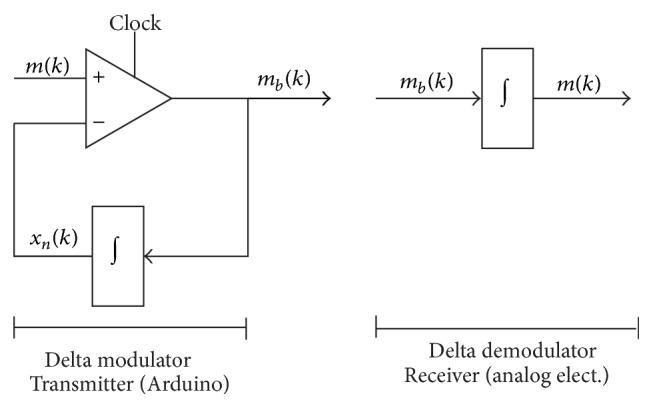
Diagram of the simple Delta modulator.

**Figure 7:**
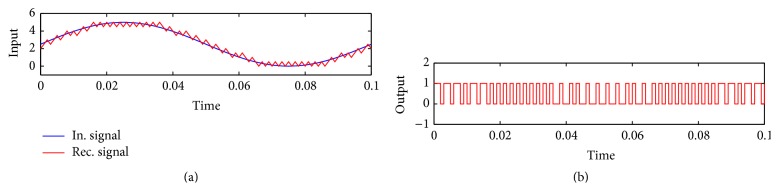
Delta modulation example of a sine input signal. (a) Input signal and reconstructed signal comparison. (b) Modulated output.

The next step is the Delta modulation. This kind of modulation can be viewed as an 1-bit ADC conversion scheme since it generates one output bit per input sample. The scheme of the Delta modulation is shown in Figure 6. It consists of a comparator in the forward path and an integrator in the feedback path of a simple control loop. The inputs of the comparator are the signal to be modulated, *m*(*k*), and the output of the integrator, *x*_*n*_(*k*). As a result, the modulated output, *m*_*b*_(*k*), is either true (high) or false (low) at any given time as shown in Figure 7. In this figure we see an input signal and the integral of the expression *m*(*k*) − *x*_*b*_(*k*). For instance, if *m*_*b*_(*k*) is ramping up and its output is less than the input, the integrator output will continue ramping up; otherwise it will ramp down. The signal *m*_*b*_(*k*) is the differential of the input and thus it can be reconstructed in the receiver by integrating it. In this work, the integral signal, *x*_*n*_(*k*), is digitally generated by the Arduino program. On the other hand, the reconstructing integrator of the receiver is implemented with analog electronics as will be explained later. A full description of the Delta modulation technique can be found in Taylor [27].

After one bit from the Delta modulator is obtained, the next step is the message encryption. In order to do so, two logistic maps are called to generate two values *x*_1_(*k*) and *x*_2_(*k*). The logistic maps have different initial conditions; that is, *x*_1_(0) ≠ *x*_2_(0). Firstly, the message is coded with a value true or false that is assigned depending on the value *x*_1_(*k*) of the first chaotic map as can be seen in Algorithm 1 (Part 1), where *m*_*e*_(*k*) is the encrypted message and *s*(*k*) is the key necessary to retrieve *m*_*e*_(*k*).

In order to increase the security of the system, the key, *s*(*k*), is further encrypted following the same scheme. It is done by assigning it a value true or false that depends on the second chaotic map value, *x*_2_(*k*), as shown in Algorithm 1 (Part 2), where *s*_1_(*k*) and *s*_2_(*k*) are auxiliary signals that are used for encrypting and decrypting the key signal *s*(*k*).

The key is then finally encrypted by applying the XOR function to the variables *s*_1_(*k*) and *s*_2_(*k*) to yield Algorithm 1 (part 3).

The signals *s*_*e*_(*k*), *m*_*e*_(*k*), and *s*_1_(*k*) are sent to the receiver through digital outputs D2, D4, and D7, respectively.

In the receiver, the signals *s*_*e*_(*k*), *m*_*e*_(*k*), and *s*_1_(*k*) go directly to the Arduino inputs D2, D4, and D7, respectively. The flow diagram of the receiver program is shown in Figure 5 as well. The first step in decrypting the message is the decryption of the key signal *s*_*e*_(*k*). This is done by applying the boolean formula that reverts the encryption. The formula to calculate *s*_2_(*k*) given *s*_*e*_(*k*) and *s*_1_(*k*) is obtained as follows. Recall that in the transmitter *s*_*e*_(*k*) is obtained by using the XOR function

(2) $$\begin{array}{c} s_{e}\left(k\right)=s_{1}^{\prime }\left(k\right)\cdot s_{2}\left(k\right)+s_{1}\left(k\right)\cdot s_{2}^{\prime }\left(k\right), \end{array}$$

where (·)′ is the complement operation of the corresponding logic variable. The truth table of the function in (2) is shown in Table 1. Thus, given *s*_1_(*k*) and *s*_*e*_(*k*) for obtaining the signal *s*_2_(*k*) would result in the truth Table 2.

The Karnaugh maps technique [34] was used to find the desired simplified expression for *s*_2_(*k*). It is a pictorial method in which the truth table of the boolean function to be simplified is represented in a bidimensional form. The boolean variables are arranged according to the Gray code. The terms of the simplified expression are found by grouping 1s or 0s in an optimal way and therefore eliminating unnecessary variables. As a result, the following boolean expression for *s*_2_(*k*) is obtained:

(3) $$\begin{array}{c} s_{2}\left(k\right)=s_{1}^{\prime }\left(k\right)\cdot s_{e}\left(k\right)+s_{1}\left(k\right)\cdot s_{e}^{\prime }\left(k\right). \end{array}$$

Once *s*_2_(*k*) is retrieved, the signal *s*(*k*) is obtained with Algorithm 2 (part 1).

The signal *m*_*e*_(*k*) is finally decrypted by analyzing the value of *s*(*k*) (see Algorithm 2 (part 2)), where *m*_*d*_(*k*) is the decrypted signal. The output *m*_*d*_(*k*) is sent to the output pin D3 and it goes directly to the Delta demodulator realized with analog electronics using operational amplifiers.

**Figure 8:**
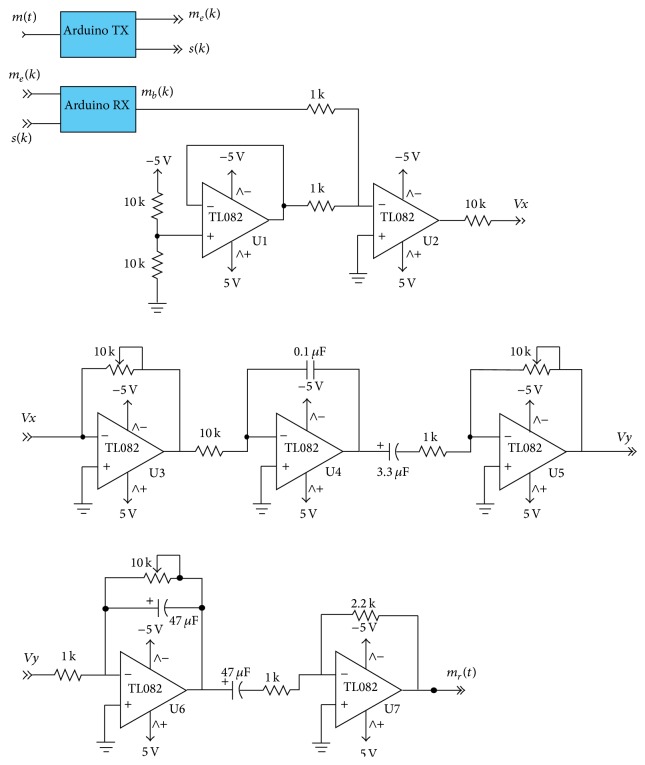
Circuit diagram of the analog electronics in the receiver.

As shown in Figure 6, the Delta demodulation consists of an integrator. The signal is passed through different stages as shown in the circuit diagram of Figure 8. The circuit has three main blocks. The first one, composed of the amplifiers U1 and U2, is a unipolar to bipolar converter. Recall that the Arduino inputs must be unipolar so in the case that the original signals are bipolar they must be recovered to its original form at the output of the Arduino. Thus the signal *m*(*k*)∈[0,5] V is converted to a signal *m*(*t*)∈[−2.5,2.5] V. The second block is composed of amplifiers U3 and U4. They are designed to compute the integral of the input signal. It consists of an integrator that performs the Delta demodulation (U4) and an inverter amplifier (U3) to adjust the quality of its output. These signals are finally sent through a low-pass filter, an amplifier, and an inverter (amplifiers U5–U7) to get the final *m*_*r*_(*t*) which should be approximately equal to *m*(*t*).

### 4.2. Experimental Results

**Figure 9:**
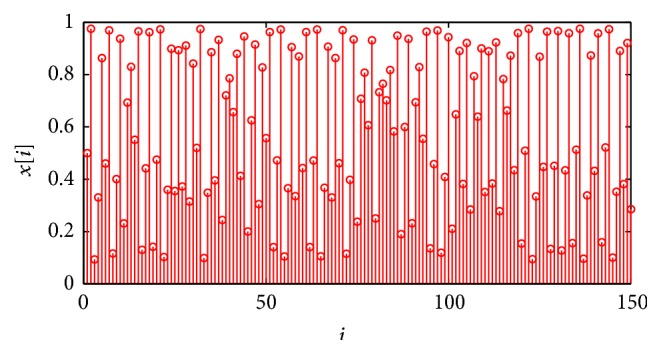
Numbers generated by the logistic map with *r* = 3.9018 and *x*(0) = 0.5.

For the experiments, the logistic maps were implemented with *r* = 3.9018 and initial conditions *x*_1_(0) = 0.1 and *x*_2_(0) = 0.5. As an example, the sequence of numbers generated by the logistic map when *x*(0) = 0.5 is shown in Figure 9. Each loop of the transmitter algorithm is executed by the Arduino microcontroller in 210 *μ*s approximately while each receiver loop is executed in 25 *μ*s approximately. This means that the message signal bandwidth should be at most 500 Hz approximately in order to be well retrieved in the receiver.

**Figure 10:**
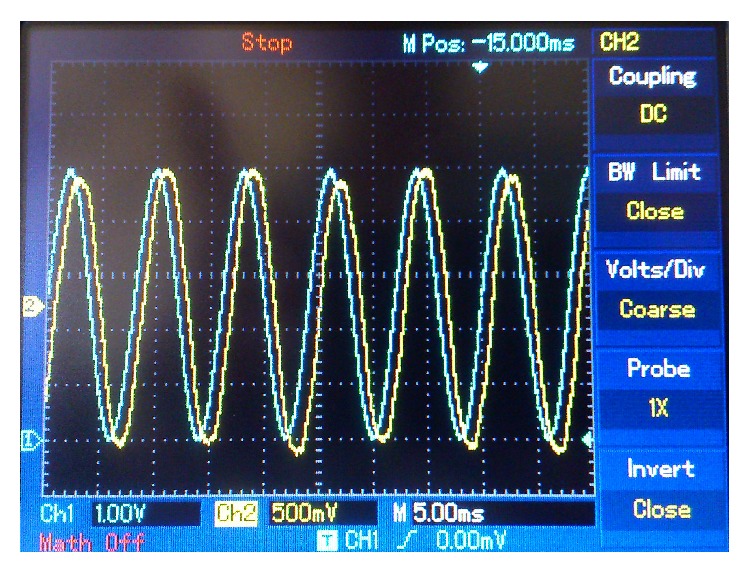
125 Hz sine wave message. Blue: sent message. Yellow: retrieved message.

**Figure 11:**
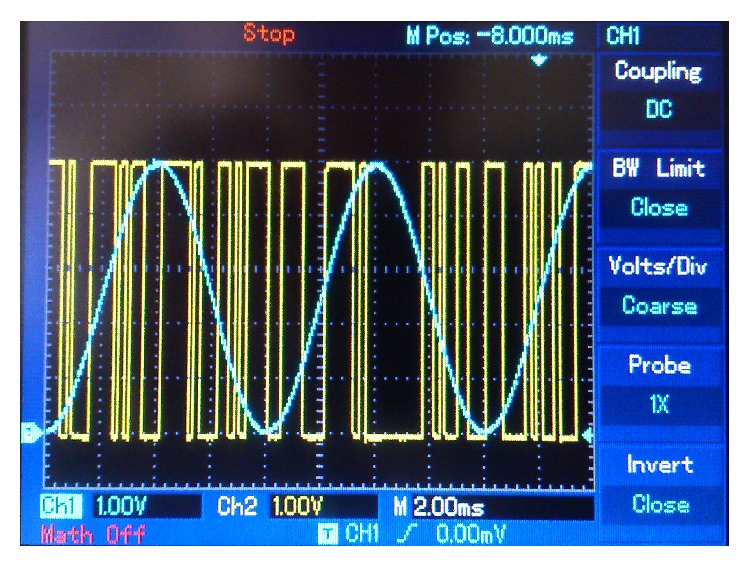
125 Hz sine wave message. Blue: sent message. Yellow: encrypted message signal.

**Figure 12:**
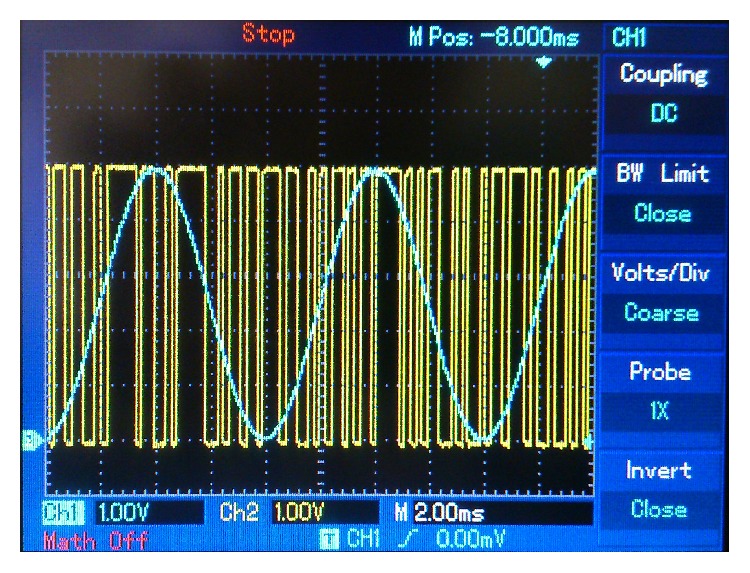
125 Hz sine wave message. Blue: sent message. Yellow: encrypted key.

**Figure 13:**
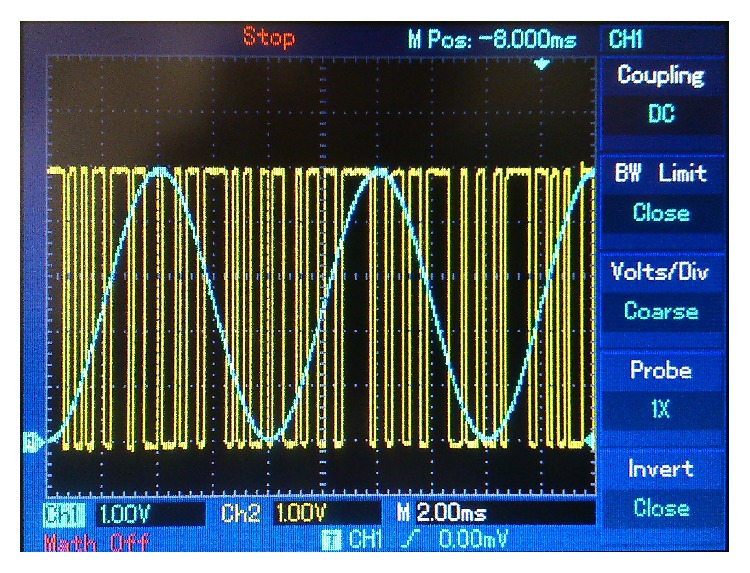
125 Hz sine wave message. Blue: sent message. Yellow: auxiliary signal *s*_1_(*k*).

Figures 10 to 13 are screenshots of the oscilloscope corresponding to the first experiment. In this case, a 125 Hz sine wave, 5 V peak-to-peak amplitude, was used as a message signal. In Figure 10 we see a comparison of the sent message, *m*(*t*) (in blue), and the retrieved message, *m*_*r*_(*t*) (in yellow).  Figure 11 compares the sent message, *m*(*t*) (in blue), and the encrypted message signal, *m*_*e*_(*k*) (in yellow).  Figure 12 is a comparison on the sent message, *m*(*t*) (in blue), and the encrypted key signal, *s*_*e*_(*k*) (in yellow). Finally, Figure 13 compares the sent message (in blue) and the auxiliary signal *s*_1_(*k*) (in yellow).

**Figure 14:**
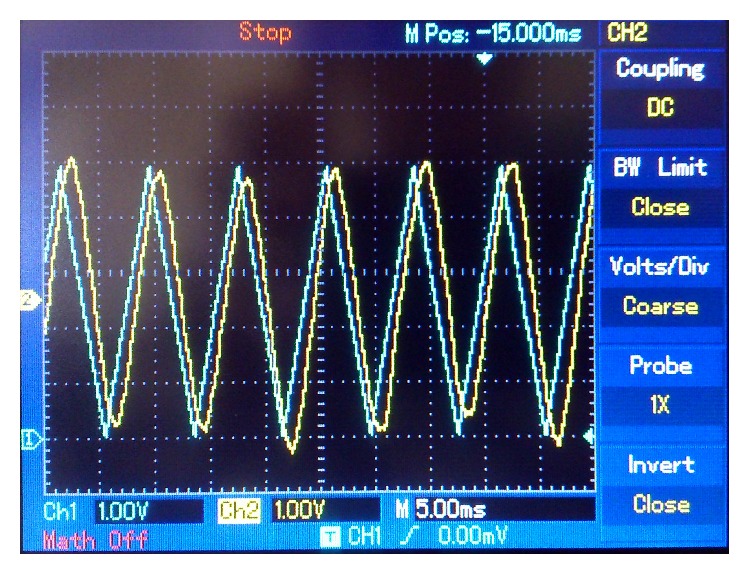
125 Hz triangular wave message. Blue: sent message. Yellow: retrieved message.

**Figure 15:**
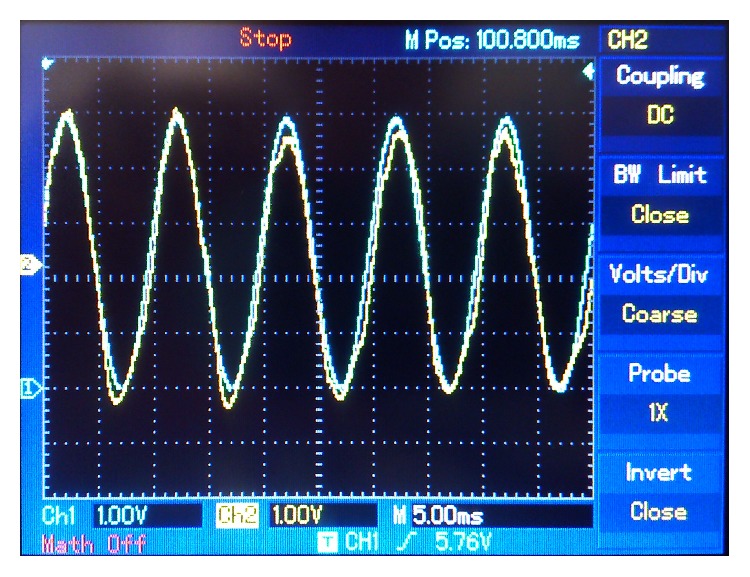
70 Hz sine wave message. Blue: sent message. Yellow: retrieved message.

**Figure 16:**
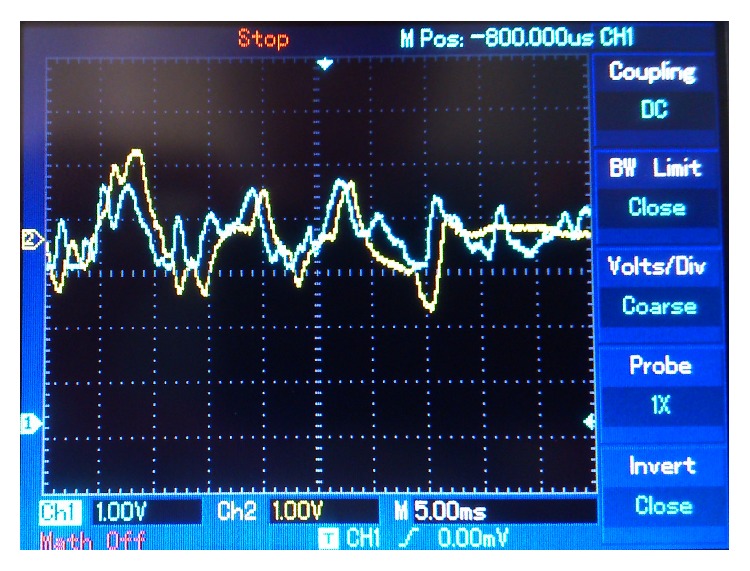
Random wave message. Blue: sent message. Yellow: retrieved message.

In subsequent experiments, different frequencies and waveforms were tested.  Figure 14 shows a 125 Hz triangular wave message (in blue) and its retrieved version (in yellow).  Figure 15 compares a 70 Hz sine wave message (in blue) and its retrieved version (in yellow). Finally Figure 16 shows a random wave message (in blue) and its retrieved version (in yellow). This signal was generated by making sounds through an* electret* microphone.

## 5. Conclusion

In this paper we presented a communication system based on chaotic logistic maps and an experimental realization of it. The proposed communication system uses a simple Delta modulator to modulate the message signal and a logistic map for encryption. A key signal is also generated and encrypted in order to retrieve the message in the receiver side without the need for synchronization. The whole system was implemented with Arduino Uno microcontroller boards that run the encryption and decryption algorithms in the transmitter and receiver, respectively. The experiment results showed the feasibility of using the Arduino microprocessors for the task proposed. With the proposed scheme, it is possible to transmit signals whose bandwidth is 500 Hz approximately.

## Figures and Tables

**Algorithm 1:**
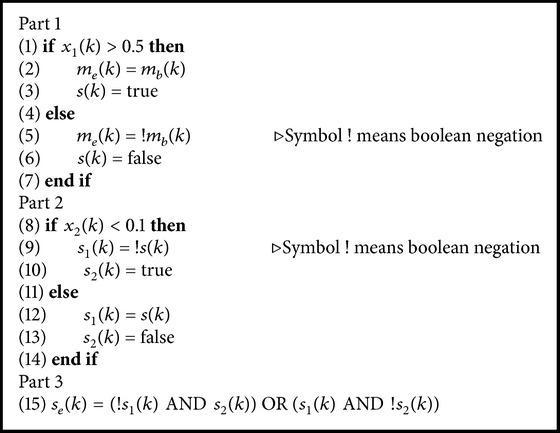


**Algorithm 2:**
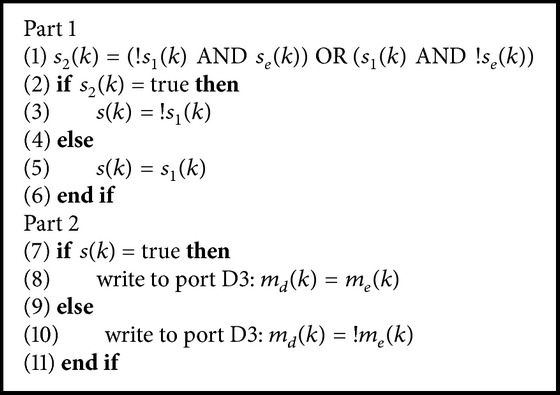


**Table 1:** Truth table for *s*_*e*_(*k*).

*s*_1_(*k*)	*s*_2_(*k*)	*s*_*e*_(*k*)
0	0	0
0	1	1
1	0	1
1	1	0

**Table 2:** Truth table for *s*_2_(*k*).

*s*_1_(*k*)	*s*_*e*_(*k*)	*s*_2_(*k*)
0	0	0
0	1	1
1	0	0
1	1	1
